# ‘Life Minus Illness = Recovery’: A Phenomenological Study About Experiences and Meanings of Recovery Among Individuals with Serious Mental Illness from Southern India

**DOI:** 10.1007/s10597-024-01312-4

**Published:** 2024-07-04

**Authors:** Srishti Hegde, Shalini Quadros, Rashmi Appaji, Vinita A. Acharya

**Affiliations:** 1 Department of Occupational Therapy, Manipal College of Health Professions, Manipal Academy of Higher Education, Manipal, Karnataka 576104 India; 2 Department of Psychiatry, Kasturba Medical College, Manipal Academy of Higher Education, Manipal, Karnataka India; 3 Present Address: Department of Psychiatry, Father Muller Medical College, Mangalore, Karnataka India

**Keywords:** Meaning, Recovery, Serious Mental Illness, Indian context, Qualitative research

## Abstract

Traditional medical models have given way to recovery-oriented approaches over the years in the management of individuals with serious mental illnesses. However, very little is known about such recovery-based models in the Indian context. This qualitative study used a phenomenological approach to explore the experiences and meanings of recovery among individuals with serious mental illness in southern India. Purposive sampling with maximum variation was used to recruit participants. In-depth interviews were conducted with ten participants, using a semi-structured interview guide. Thematic analysis resulted in three themes: “The illness journey,” “Life minus illness = Recovery,” and “It takes a village to recover,”. Illness and recovery seemed to be two sides of the same coin with the context playing an influential role in the perceptions of recovery. The term “recovery” seemed to be a misnomer giving the impression that one is expected to return to an illness free state.

## Introduction

Serious Mental Illness (SMI) is any mental illness that hinders with a person’s life and ability to function, some of the common diagnoses included being schizophrenia, major depressive disorder and bipolar disorder (Substance Abuse and Mental Health Services Administration [SAMHSA], 2022). These illnesses are considered to be severe, persistent, and life-long due to the nature of their deficits and their ongoing influence on the individual’s functioning. Traditionally, clinical, or functional recovery and rehabilitation efforts have emphasized symptom reduction, remission, decreased hospitalization, the ability of the individual to return to educational or employment activities, have meaningful relationships, and perform daily life activities necessary for independent living (Andreasen et al., 2005; Law & Morrison, 2014; Silverstein & Bellack, 2008).

However, psychosocial rehabilitation has seen a gradual transition from the traditional medical model to a recovery-based model (Jacob, 2015). Recovery refers to a process of learning how to manage daily life in the presence of, or within the limitations imposed by the mental illness. Personal recovery comes from narratives of people living with mental illness and is thus more individualized (Davidson et al., 2021; Harvey & Bellack, 2009). Therefore, the term recovery in mental health care can be understood as an individual’s journey of enhancing their health and well-being, living a self-directed life, and working towards achieving their full potential (SAMHSA, 2018).

In 2011, Leamy et al., synthesized existing recovery literature into an empirical conceptual framework, including recovery processes such as Connectedness, Hope, Identity, Meaning, and Empowerment (CHIME). The perspectives of recovery and what it meant are allowed to differ from person to person (Mathew et al., 2018). In this recovery-oriented approach the responsibility is perceived to be shifted completely on the individuals with mental illness and away from services. The service users also felt that the approach of professionals was not conducive to recovery and that their professional training made them perceive mental illness in a generic way (Aston & Coffey, 2012).

The progression of slowly accepting oneself and one’s illness and moving on to live a life beyond the illness has often been metaphorized in literature as a ‘journey’ (Durgu & Dulgerler, 2021; Vaingankar et al., 2020). Wood and Alsawy, in 2018, conducted a systematic review of qualitative studies in which personal agency, the need to accept self, be empowered, having a routine, and set achievable goals have been found to be salient for recovery. Literature about recovery has also highlighted the importance of social and emotional support of families, and communities in the individual’s recovery journey (Petros et al., 2016; Vansteenkiste et al., 2021). Social deprivation, negative social relationships, substance misuse, negative influence of medicines and stigma on the other hand, were considered as barriers to recovery (Hancock et al., 2018; Wood & Alsawy, 2018).

Engaging in everyday activities or occupations such as academics, work, recreation, being part of festivities, and spending time with loved ones are known to foster recovery (Clarke &Warner, 2016), while lack of skills, peer support, and limited opportunity and choice for engaging in occupations were seen as barriers to recovery (Kelly et al., 2010). Occupational therapists are professionals trained to understand how mental illnesses affect the functioning of individuals and work towards facilitating participation in the same (Krupa et al., 2009). Due to this congruence to the recovery paradigm, occupational therapy has frequently been recognized as the ideal facilitator of this paradigm shift from traditional rehabilitation model to a recovery based one (Nugent et al., 2017).

Perceptions about recovery have been found to be varying with differences in contexts as well. Only two Indian studies (Gandhi et al., 2020; Gopal et al., 2020) were found to be related to recovery perspectives. They reported the absence of symptoms, being independent, working, getting married and raising a family as important aspects of recovery. Apart from the factors seen in the Western studies, the Indian studies have also placed more emphasis on factors such as family, spirituality and relationship with God and their influence on recovery. The role of family, friends, community, and spirituality have been reported as strong facilitators of recovery in studies from collectivist cultures (de Wet et al., 2015; Durgu & Dulgerler, 2021; Ricci et al., 2021; Subandi, 2015; Vaingankar et al., 2020).

In India, mental health care is still close to the traditional model and many professionals continue to prioritize clinical or functional recovery in practice. The concept of recovery and its implementation in practice, are better studied in mono**-**cultural, English-speaking countries, with individualistic attitudes and a strong sense of independent personal identity. Although recovery is influenced by the culture and values of the individual and the community they belong to, few studies have highlighted the meaning of recovery in non-Western countries such as India, with collectivist attitudes, varied socioeconomic conditions, diverse culture, and a sense of community identity. Considering that India is a diverse country with additional challenges such as low literacy levels and stigma related to mental illness, it may not be feasible to assume findings from the developed Western or European countries could be easily applied to the Indian context (Dsouza et al., 2017; Gaiha et al., 2020; Murthi & Hammell, 2021). It is always recommended that new knowledge is generated from the target context to facilitate a better understanding of the issue at hand. Hence our specific research questions were ‘*What are the experiences and meanings of recovery among Indian individuals with SMI?’* and *‘What role do activities and occupations play in the process of recovery of individuals diagnosed with SMI?’.*

## Materials and Methods

### Study Setting

The participants were recruited from the outpatient departments of two tertiary care hospitals, that were multidisciplinary in nature. Individuals here were first seen by psychiatrists, who led the team and were later referred to other services such as psychology, social work, and occupational therapy as per need. Individuals from in and around Karnataka, of various socioeconomic, and educational backgrounds are known to come here for consultation on inpatient or outpatient basis.

### Study Design and Participants

In this multi-centric, qualitative study, we used a descriptive phenomenological approach to understand the experiences and meanings of recovery among individuals with SMI. We chose this research design as it places heavy emphasis on the lived experiences of people while encouraging the researcher to adopt an open stance, question their prior understandings, and assume a reflective attitude (Sundler et al., 2019). Individuals between the ages of 18 and 59 years, male or female, speaking English or Kannada, diagnosed for a period of at least two years with a SMI were included in the study. Individuals who had a dual diagnosis or a diagnosis of substance use were excluded from the study. As the researchers were keen to understand the various experiences of individuals, the maximum variation sampling technique of purposive sampling was used. This allowed us to construct a sample by identifying crucial dimensions of variation and recruit individuals who varied from each other as much as possible. Using this technique helped to have a holistic understanding of the phenomenon as experienced by individuals of varied contexts (Suri, 2011).

### Ethical Considerations

Following approval from the Institutional Research Committee, ethical approval was sought from the Institutional Ethics Committee (IEC 171/2022) and registration was completed under Clinical Trials Registry of India (CTRI/2022/09/045435).

### Data Collection

**Fig. 1:**
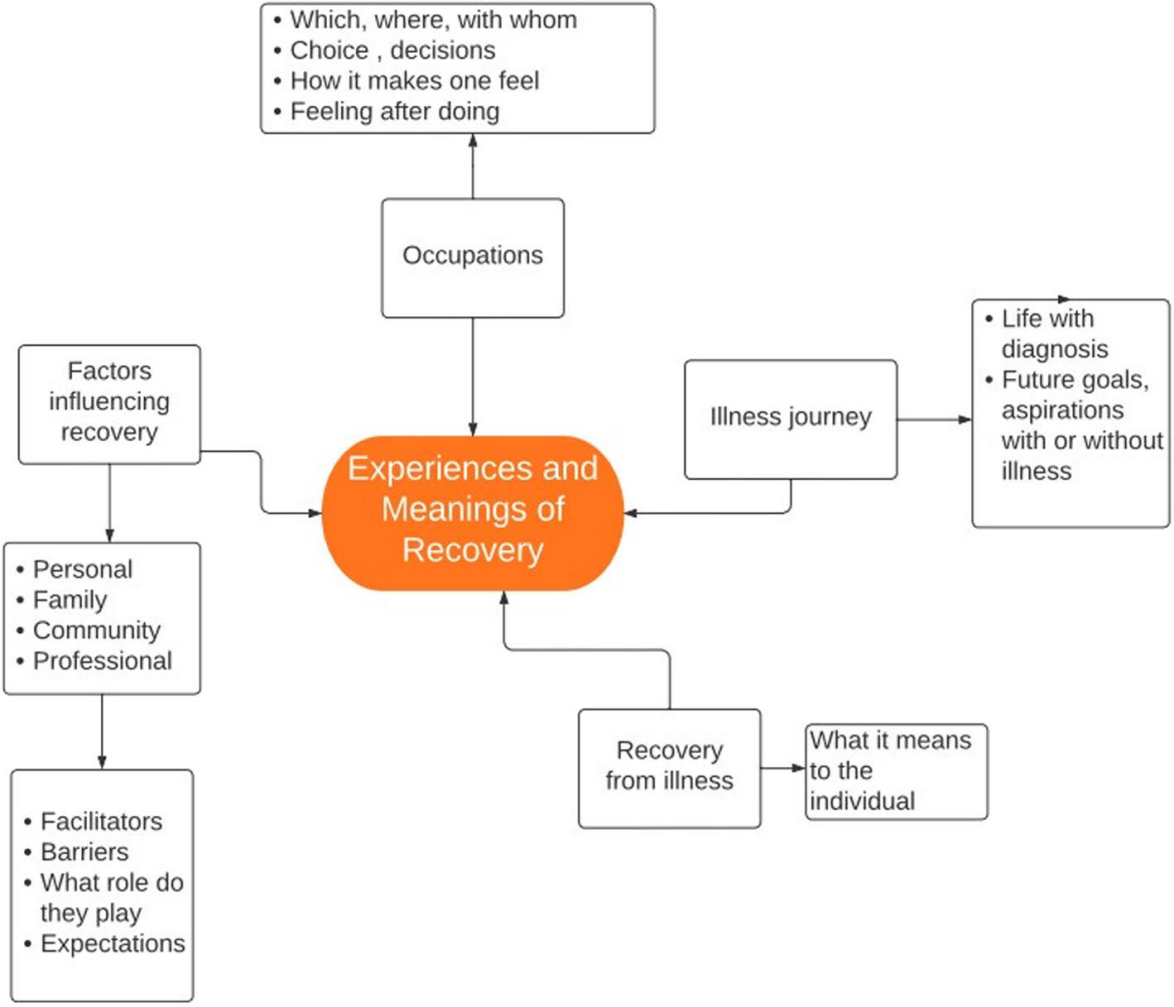
Conceptual framework

A conceptual framework (as seen in Fig. 1) was developed based on literature review, and discussions on existing concepts in the field of recovery. Based on this an interview guide was formulated containing questions such as, ‘*What do you understand by the term recovery?’,* ‘*How far do you think you have come on the path of recovery?’,* and ‘*What are some of the factors that you think have influenced your recovery?’.* These were then validated by two experts who had prior experience in qualitative research methods as well as mental health research. Necessary modifications were made to the interview guide based on their feedback.

A total of ten participants were interviewed as part of the study. Table 1 describes the characteristics of the participants. A list of potential participants, meeting the inclusion criteria, from the patients present at the outpatient department was made after discussion with the concerned psychiatrist. The psychiatrist would opine if the patient could be considered for the interview based on their clinical status. Stable patients, as recommended by the psychiatrist, were then approached for recruitment. The study was explained to potential participants using the participant information sheet. Written and verbal informed consent were later obtained from interested participants, willing to be a part of the study. Most of the participants were not familiar with the researcher, however, were comfortable as they were spoken to in a language familiar to them. Efforts were made to reassure them, provide detailed information to clarify any questions they may have, and also provided opportunity to refuse if they did not wish to participate. During the study period, four patients had refused to participate, one as she was hesitant and unsure of participating, while the other three refused due to logistic reasons. They could not stay back for the interview as they had come from far and had to leave after the consultation to reach home in time. The primary researcher who was bilingual and fluent in English and the local language conducted in- depth, face -to-face interviews of about 45–60 minutes with the participants who consented to be part of the study. In-depth interviews allow the participants to explain in their own words, how they interpret situations, experiences, and the world around them (Knott et al., 2022). Hence, this was considered to be a suitable method for data collection as the study aimed to understand the personal experiences of individuals with mental illness about recovery. Eight out of the total ten interviews were conducted in *Kannada*, the local language, as it was preferred by the participants, while the other two were conducted in English. All interviews were conducted in a private room within the Department of Occupational Therapy, to ensure participant comfort and privacy. Interviews began with broad open-ended questions about the individual’s illness journey and future aspirations. Every question was explored further using prompts and probes asking for more detailed explanations or information regarding their views*.* Following each interview, experiences and reflections were discussed by the first author with the corresponding author, which aided in modifying certain questions of the interview guide. Since no new information was coming up, most of the data was repetition from previous interviews and as it was a time bound study, data collection was concluded after completing ten interviews. All the interviews were audio recorded and then transcribed (transliterated first and then translated if done in Kannada). Confidentiality was maintained, participants were kept anonymous and fictitious names were used during writing. All recordings were maintained safely with the primary researcher and the corresponding author.

**Table 1:** Participant characteristics

Participant (Fictitious names)	Gender	Age	Diagnosis	Duration of illness (years)	Marital status	Education	Occupation	Socio-Economic Status	Living Arrangement
Sudha	Female	52	BPAD^a^	27	Married	Primary school	Homemaker	Low	With family
Chintan	Male	20	RDD^b^	6	Single	Undergraduate	Student	Middle	With parents
Sharath	Male	27	BPAD	14	Single	Undergraduate	Farmer	Middle	With parents
Naveen	Male	36	BPAD^a^	20	Married	Primary school	Labourer	Low	With family
Vrinda	Female	50	BPAD^a^	10	Single	High school	Homemaker	Low	With mother
Vijay	Male	34	Paranoid Schizophrenia	15	Single	Undergraduate	Medical Intern	Middle	College hostel
Meena	Female	33	BPAD^a^	19	Married	Undergraduate	Cashier	Low	With husband
Sandhya	Female	33	BPAD^a^	12	Married	Higher secondary	Homemaker	Middle	With family
Nirmal	Male	52	Paranoid Schizophrenia	27	Single	Undergraduate	Office boy (currently not working)	Middle	Hostel
Suman	Female	40	Paranoid Schizophrenia	20	Married	Undergraduate	Homemaker	Middle-High	Rehabilitation facility

^a^BPAD: Bipolar Affective Disorder; ^b^RDD: Recurrent Depressive Disorder

### Data Analysis

Thematic analysis was used to interpret and analyze the data gained from interviewing individuals with SMI. All transcripts were read and re-read by the first and corresponding author, to gain familiarity. The data was searched for meanings and these meanings were marked as codes. The codes were then further organized into categories. Coding and categorization were done for each transcript independently and then discussed between the key team members. Categories were later further arranged into patterns that were related to each other and combined to form themes. Microsoft Excel was used to manage the data during the analysis. The themes were given creative yet clear names to capture the essence of their content (Sundler et al., 2019). Trustworthiness of the study was ensured through frequent debriefing and reflective discussions between the research team members, along with audit trails that were maintained.

## Findings

**Fig. 2:**
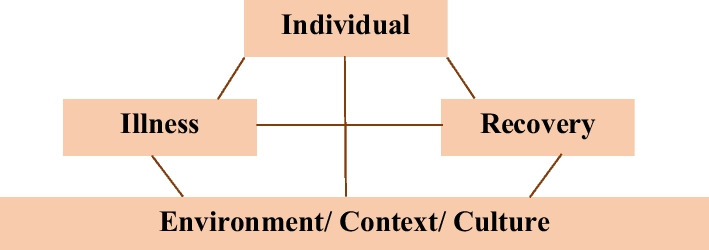
Diagrammatic representation of the relationship between themes

Three themes emerged from the analysis, which were as follows: **‘The illness journey’, ‘Life − Illness = Recovery’,** and **‘It takes a village to recover’**. Table 2 describes the sample codes and categories in each of the themes. Figure 2 represents a proposed relationship between the themes where the individual experiences mental illness and recovery as two sides of the same coin; one cannot exist without the other. They each take turns to ebb and flow in the lives of individuals with mental illness. Environment or context forms the base, the foundation for and within the limitations of which, these interactions are at play.

**Table 2:** Illustrative example of thematic analysis

Sl.no	Themes	Categories	Codes
1	The illness journey	Experience of illness	Why illness, feeling numb, anger outbursts, no motivation
		Medication: A hard pill to swallow	Initial non-compliance, medications affect routine, grateful for medications
		Impact of illness: The damage done	Could not study further, social circle affected due to illness behaviour, no courage to think about future with illness
2	Life minus Illness = Recovery	Recovery: What it means	To be cured, to be well, illness free, come out of illness
		Doing drives recovery	Work helps to engage mind, contributing to family, hobbies, socialising, sitting idle makes illness worse
		Believing in the beyond	Belief in God, having hope
3	It takes a village to recover	Circle of family and friends	Lives with family, family encourages taking medicines, family provides reassurance
		Professionals as guides	Professionals give medicines, professionals provide guidance
		The larger community	People make hurtful comments, unnecessary advice, age is debarred

### Theme 1: The Illness Journey

This theme describes the illness related experiences of the participants, their relationships with medications and the impact that mental illness seemed to have had on their lives. All of these, in turn, appeared to have influenced how they perceived recovery.

***Experience of Illness:*** They reported being uncontrollable during periods of illness, often causing a scene, getting angry, talking excessively, being impulsive and behaving unusually. They seemed to struggle without answers to the question, “Why me?”.

One participant compared her symptomatic phase to a stone, describing an absolute numbness where nothing mattered to her, calling it the “silent period.” She also shared how soon after this phase of silence there would be a phase of extreme energy where she would believe she could achieve anything. She found it hard to believe that even these phases of seeming empowerment were not healthy. She feared that the courage she thought she gained during one of these active phases would go away with medications causing her to be even more confused about who she truly was. She expressed her dilemma as follows:

*“When I have so much energy, I feel like I can do anything…, is it the strength God gives me or the strength this illness gives me? when I take medications, I no longer have that courage…”*

*“…but when I take medications, I have no belief in myself…” *(Sudha)

***Medication: A hard pill to swallow:*** Participants expressed a constant ambivalence towards medications, often unsure if they shouldn’t mind taking them for the benefits, or dislike them for all their side effects. Many of the participants reported not being compliant with medications in the initial phase of their illness and often had to be given medications without their knowledge or had to be forced to take them. A few participants shared their reservations of having to take medications forever.

*“I don’t want to be sick again and again…we need to keep taking medications, right… [that] I feel bad… [about]…”* (Vrinda)

Some of the participants had often stopped taking medications either because they were feeling better or on the misguided advice of family and friends that these were harmful to them. Although many of the participants admitted to the side effects such as feeling drowsy or unable to wake up early in the morning, and weight gain, they continued taking them, out of fear of a relapse. As one of the participants reported,

*“When that happened in 2014 [referring to a relapse] …I had stopped my medication for one day…before that I had taken regularly…but still it happened, I don’t know how…”* (Sharath)

*“I am able to work because I am swallowing tablets…whatever I do…I take my tablets…”* (Naveen)

They seemed to be resigned to the fact that medicines were there to stay in their lives.

*“…you can taper the medications but you cannot leave them… whatever you want to do, have a routine, with medications we should do the things that make us happy…”* (Suman)

***Impact of Illness: The damage done*** Participants believed they made many compromises in life due to their illness. Many were left feeling like they were restricted to odd jobs and that they had achieved nothing. The illness not only affected the individual’s productivity but also their social circle. Participants felt that their behaviors in times of illness often affected their relationships with friends and family.

*“If I get free from this [referring to the illness], I can think about it… [a relationship] if I don’t get free from this…I don’t have the courage to think.” *(Sudha)

Many of the single participants were worried about who would look after them if they did not find anyone. The idea of having to be without a partner seemed to be a preoccupation for some of the participants. One of them said,

*“…right now, my parents are with me at home…so no problem…after they age more…who will check on me…”* (Sharath)

### Theme 2: Life Minus Illness = Recovery

This theme elucidates what participants understood by the term “recovery,” aspects that helped them in this process and those that made it difficult to recover.

***Recovery: What it means*** Recovery looked different to different participants. Some chose to describe it in terms such as being active, happy, healthy, brave, having a routine, being able to look after themselves, socialize, and help others. One of the participants said,

*“Recovery means…to get better…to adjust with people …like that…to mingle…to be adjusting at home…to be occupied in different kinds of work…and to have these hobbies.”* (Vrinda)

Some participants used the term “normal” to describe recovery, indicating the absence of illness, reduction in symptoms or no longer needing doctors. The term “recovery” was often understood literally as to be cured. According to one participant, the term “recovery” was being incorrectly used in psychiatric illnesses, as they are usually long term. He said,

*“In mental health condition…one who has entered in psychiatry department as a patient it is very difficult for him to come out of this zone…all the time if he comes out of one problem, he will be trapped in another problem … here recovery is not permanent …it is temporary…so recovery is a wrong term in psychiatry…never.”* (Vijay)

While some participants described themselves to be “recovering,” implying it was a process and there was a long way to go, some others, on the other hand, thought of recovery as a complete absence of symptoms, which was never likely to occur.

*“It is difficult to say recovery is a process…like what I have, it is difficult to recover from that…how much I have understood, even if we take medicines this may not be controlled…”* (Sharath)

***Doing drives recovery:*** Many of our participants reported that “doing” made them happy and feel better. Activities of their interest especially had the ability to change their mood and facilitated good mental and physical health. Participants listed a range of activities and hobbies such as gardening, yoga, traveling, watching movies, and journalling among others. As one of them said,

*“I like gardening, especially flowering plants…it makes me happy, when the flowers bloom it is nice to see…I feel, “I have done that… I grew it and now it has flowers…”* (Sandhya)

For some, meditation helped reduce confusion and gain clarity. Doing seemed to distract them from negative thoughts. Participants judged their own recovery by how much they were “doing,” and what they could not or were not able to do. Being around people, spending time with loved ones and talking to friends helped most participants to feel better. Two of the female participants also shared how nurturing their family, taking care of their spouse and children was a constant motivator.

*“…and spending time with my children helps… when I speak to them…I feel better… They come talk to me…they say Amma [mother] do this …do that…it is nice…”* (Sandhya)

Being able to work played a very important role in their recovery. Earning and contributing to the family was a central indicator of recovery to the participants.

*“…when I earn, I feel like I put effort in getting that money…I can save, I can help my parents, that gives me a lot of satisfaction…”* (Meena)

On the other hand, inability to earn made another participant feel like she had no right to have an opinion or say in her family. She said,

*“I cannot say anything (chuckles)…if I was working, I could have given them some money…now I don’t have anything…I have to listen to them…”* (Sudha)

One of the participants who worked as a daily wage worker took pride in his ability to work and put food on the table for his family of five. He shared,

*“If I did not earn then…it was very difficult for me…because of that only …I am earning now and able to put food on the table…back then …some days we would eat just ‘ragi mudde’ [basic staple food in the region] and chutney. Now we get rice [considered a luxury] from the shop…because I earn, I am able to buy things from the shop for the house…that is all.”* (Naveen)

Participants also highlighted not having anything to do made their illness worse, causing them to fixate on unnecessary negative thoughts.

***Believing in the beyond:*** Many of the participants relied on spirituality and took solace in their faith in God to face their everyday hardships. While some believed recovery was God’s will, one believed it was a partnership. He said,

*“…it goes on…nothing is in our hands. Whatever is supposed to happen…will keep happening…some things are in our hands. Any work…80% it’s on us…20% it’s on God… whatever it is…be it job… now like for me …I wrote CET [Common Entrance Test] …in that 80% was my effort…20% …left to God.”* (Sharath)

### Theme 3: It Takes a Village to Recover

***The circle of family and friends:*** Family seemed to be an important aspect of the social fabric for the participants. Most of the participants at the time were living with their family, either with their parents or with spouse and children. One of the participants expressed how there was nobody in the world who would care for him the way his parents had been doing. He said emotionally,

*“…. the way your parents check up on you…others cannot…they have to bring you here [to the hospital] …all that is very difficult.”* (Sharath)

Two of the women expressed their gratitude for the support of their husbands, for taking up more than their share of responsibilities when they were ill. For some participants, apart from families it was their friends, neighbors, and colleagues who had been accommodating and motivating. At times, it had been random people in the community, in the form of the pastor, helpful people at the bank and strangers who found them and brought them to hospital in times of distress.

However, for some participants, they hardly got any support from their families and were almost abandoned. Participants reported feeling emotionally disconnected with their families, and often isolated and lonely. Others shared feeling like victims due to hurtful comments and labels, leaving participants with little or no networks and social connections.

*“When I was in the hostel…I fought with the warden…he said “I’ll throw you out of the hostel, you are a patient.” *(Sharath)

***Professionals as guides:*** Participants valued the assurance and guidance of their doctors. This was vital for those who had no other real connections in society or with their families to rely on when in confusion.

*“I know a lot of people…but cannot call them friends, I don’t talk freely to anybody…I message sir [his doctor] whenever I have a problem…”* (Nirmal)

Another participant expected professionals to assist in getting through to family members regarding decisions he was not confident to voice himself. Some shared how they needed someone to take time and talk to them.

***The larger community:*** In general, in the society, participants experienced people to be judgmental and stigmatizing. According to the participants, people assumed mental illness to always be “disabling.”

*“…in small town you don’t know about any …particularly about mental illness. They are talking with me like I am mentally retarded person. Sometimes they think that I am not capable of doing things…” *(Chintan)

Some participants felt that there were social milestones to be achieved as per their age. Participants past the age of 30, who were unmarried seemed to have resigned to the fact that they had gone past the deadline to get married or have children. This had a serious implication to how they thought about their recovery.

*“…earn on my own…look after my wife, children and all…that is my dream, but my age is debarred … so now even if I earn, I will not have children and all at the age of 53….”* (Nirmal)

## Discussion

Our study aimed to explore the meanings and experiences of individuals with SMI regarding their recovery. Our participants understood the term “recovery” quite literally, meaning to be cured and being illness free, which kept them tethered to the medical connotation. As mental illness was thought to be permanent, needing lifelong medications, the term was thought of as a misnomer in psychiatry. This is in line with other studies where individuals have found the term to be confusing, mechanical and that which indicated that something was broken and needed to be fixed (Mccauley et al., 2021). Aston and Coffey (2012), as part of their study asked their participants to suggest alternatives to this term, to which they came up with words such as “spectrum”, “cycle” or “half recovery” which showed the fluctuating, repeated and incomplete experiences of individuals. While reflecting on our participants’ responses, and looking for a suitable alternative to the term “recovery,” the concept of Kawa by Michael Iwama seemed to fit best in this context. The Kawa model, visualizes the flow of the river to be the flow of life, and the rocks as hurdles (Iwama et al., 2009). Thus, leading us to the word “flow” as an alternative to describe recovery, to mean that the flow of life should not be impeded by the illness, but that with support one can learn to flow over and beyond one’s hurdles.

Though some of our participants described their efforts in trying to build a life beyond the illness, they have not recognized this as “recovery.” Concepts from the CHIME framework by Leamy et al. (2011) seemed to be only partly reflected in our findings. Connectedness was valued by the participants but not always experienced in their relations with family and friends. Hope was harnessed through faith and spirituality, with the aspiration of going back to ‘normal’ lives. While this could also be due to the hospital environment within which we conducted our interviews, individuals seemed to have deeply internalized the illness as their identity. In our study, participant’s visualizations of recovery were limited to everyday activities, or what Vaingankar et al. (2020) called, “hidden in ordinariness.” Participants did not want to be different, they aspired to be just like everyone else, ‘without illness’. Individuals found their everyday lives, roles, and responsibilities meaningful. Empowerment, however, did not come up too strongly in our findings. Western literature places a lot of emphasis on individualism, autonomy, independence, and the notion of a productive citizen, while recovery in low- and middle-income countries seems to be embedded in family, social connectedness, and interdependence (Aldersey et al., 2017; Gamieldien et al., 2021). In collectivist countries such as that in India, it is understandable that individuals tone down their sense of personal agency and have a more external locus of control (Joshanloo, 2022). Thus, individuals in this context might not feel the need or may not have enough resources and opportunity to be self-reliant (Gopal & Henderson, 2015). The conceptualization of recovery may need to be broad enough to accommodate such variations from diverse global contexts.

In our study, individuals appeared to have the illness as their identity. As the individuals associated recovery to being “like everyone else” or “normal,” taking medications on a long-term basis felt atypical to many individuals, reminding them constantly that there was something wrong with them. Similar thoughts were echoed by participants in the study by Brijnath (2015). Many participants in our study were taking medications due to the fear of relapse and hoped that if taken regularly the dosage would be tapered eventually. This might be due to a risk–benefit approach as the benefits of taking psychotropic medications outweigh the possibility of relapse (Grover et al., 2014). Studies have suggested that side effects, lack of insight, long treatment durations and stigma were often reasons for non-compliance (Selvakumar et al., 2018; Semahegn et al., 2020). Thus, it becomes crucial for us to educate individuals about the illness, medications, and their side effects in order to enable them to accept and attempt to live a life beyond their illness. A positive attitude towards medications and seeing them as an aid towards recovery would be more helpful.

One of the significant findings from our study was the relation between “doing” and recovery. Doing things was not only an important factor that contributed to recovery but was also evidence of recovery itself. Recovery was visualized as being active, happy, healthy, brave, having a routine, being able to look after themselves, socialize and help others. This is congruent with the findings of other studies which have also highlighted the importance of doing (Gopal et al., 2020; Vaingankar et al., 2020). Studies have shown how engaging in everyday ordinary occupations and work facilitates recovery by providing a sense of structure, boosting one’s social connections and an opportunity to re-evaluate one’s capabilities. It allows the individual to have meaningful experiences that empower them to build a sense of self and identity while providing hope (Doroud et al., 2015). Being able to hold on to jobs, and working efficiently is an important contributor to self-worth and confidence (Loganathan & Murthy, 2011). Considering the therapeutic benefits of work, more efforts need to be focused on providing individuals with the opportunities to work, through evidence-based approaches such as Individual Placement and Support (IPS) (Drake et al., 2016; Jagannathan et al., 2020). This is especially needed in the unskilled sector, as many individuals with mental illness have not had the opportunity to complete primary or professional education (Breslau et al., 2008). Occupational therapists with their focus on work participation and engagement can play a vital role in helping individuals develop skills required for jobs and to hold on to secured jobs (American Occupational Therapy Association [AOTA], 2014).

Hancock et al. (2018) described how most of their participants with mental illness struggled to find safety, security, housing, food, and basic necessities. This was not the case in our study as most of our participants lived with their families, either parents or spouse and children. Traditionally, families are a central institution in the Indian culture that values caregiving and emotional interdependence (Avasthi, 2010). Families are often responsible for providing financial support as part of caring for the individual, at times restricting themselves to, only that. Poverty is known to increase the risk of mental illness and relapses in low- and middle-income countries (Lund et al., 2010), thus having financial support in the form of families, protects individuals and facilitates recovery.

Negative experiences with families, on the other hand, can be a barrier to the individual’s recovery. This is consistent with the findings of other studies (Durgu & Dulgerler, 2021; Gandhi et al., 2020). High expressed emotion on part of caregivers or family members has been known to cause relapses in individuals with mental illness. Therefore, educating family members becomes essential while caring for individuals with SMI (Amaresha & Venkatasubramanian, 2012; Sadath et al., 2019). Co-occupations are known to involve a sense of shared physicality, intentionality, emotionality and meaning for individuals (Pickens & Pizur-Barnekow, 2009). Therefore, enabling individuals and family members to perform activities together would provide an opportunity for stronger emotional connection. Empowering the families by providing financial support through government schemes (Singh, 2019) to compensate for the loss of breadwinner or to address the additional expenses of caregiving may help ease some of the emotional distress of the family members.

Finding a job and keeping it, getting married and having children and having socio-economic stability are cultural milestones that are much valued by Indian individuals, often described by using the term “settled life,” (Brijnath, 2015; Gopal & Henderson, 2015). Therefore, failing to fulfil these life roles at an age-appropriate time is extremely stigmatizing for individuals, and can have a negative influence on their perception of recovery. In some situations, marriage and childbearing are considered a social remedy to the person’s “emotional distress,” (Loganathan & Murthy, 2011). These aspirations or desires of individuals need to be handled sensitively. Though marriage has been known to protect individuals, by providing a sense of support, it could also be detrimental for both individuals involved with an added risk of relapse. Individuals and their families thus, need to be informed about the complicated and multifaceted nature of the same (Srivastava, 2013). Faith in God and spirituality seemed to have provided individuals with a sense of hope and security. It is often noted that individuals who are religious, or those with a fatalistic attitude or external locus of control find it easier to make peace and accept hardships associated with their mental illness (Joshanloo, 2022). Similar findings have been found in other recovery literature (Brijnath, 2015; Clarke & Warner, 2016; Juan et al., 2021; Vaigankar et al., 2020; Vansteenkiste et al., 2021; Wood & Alsawy, 2018).

Overall, findings from our study suggest that our participants were not particularly familiar with recovery-oriented practices. They seemed to focus on the medical aspect of their condition more than a recovery perspective. Chang et al. (2021) have listed limited policies, inadequate organizational support, lack of understanding of recovery among professionals and stigma as few of the barriers in implementing recovery-oriented services. This is likely to be true for the Indian context as well. In India, there still exists paternalistic attitude in medical care, that puts doctors on a pedestal and individuals have little intent or part in decision making (Gopal & Henderson, 2015). There exists a treatment gap of almost 74% in treatment of severe mental disorders (Gururaj et al., 2016). Yet, currently, only about 0.04% of the overall national health budget is allocated towards mental health (Jhakar, 2022). Occupational therapy is a rehabilitation profession whose principles are closely aligned with those of the recovery paradigm which makes the profession an integral and essential part of a recovery oriented mental health team (Gruhl, 2005). Yet very few of our participants were exposed to rehabilitation services like occupational therapy. This highlights the need for a collaborative team effort in the rehabilitation of individuals with mental illness. In the study Ricci et al., (2021), participants expressed how medicines were helpful but not enough in facilitating recovery, they emphasized the need for occupational therapy, meditation, and other body-oriented activities among other services. Many of the participants that were a part of our study had not been referred to occupational therapy prior to this. Currently in India, occupational therapists have not even been recognized as part of the mental health service delivery team in the Mental Health Act, 2017 (Ministry of Law and Justice, 2017). Considering that occupational therapy has a lot to offer in terms of facilitating the reintegration of individuals into the community, it is disappointing to note that these possibilities are not yet recognized in this context (Samuel & Jacob, 2017). Unless mental health professionals adopt a recovery perspective, it seems far fetched that individuals with mental illness can look at themselves beyond the label of a diagnosis.

The findings from our context echo those that exist in previous recovery literature, but also suggest that recovery as it is may not be directly applicable to our context. One needs to have an added understanding of the people and their cultural beliefs and values. Mental health professionals need to first understand recovery and then assimilate it in the light of our socio-cultural milieu. Recovery, even if not coined or understood as that, is still being experienced by many individuals at various levels, but a greater sense of liberation might be felt if we could help them shed this identity of a “patient.”

### Strengths of the Study

It is one of the few studies from the Indian context that explores the perspectives of individuals with SMI about recovery. As the interviews were conducted in the local language, we were able to recruit individuals from a socio-economically diverse population. The findings are not only in line with other existing studies in this field but also additionally provide valuable insight into the collectivist and cultural background of India.

### Limitations of the Study

As India is a very culturally diverse country, the findings from this one region might not be transferable and should be considered with caution. There were no participants from higher socioeconomic background or with post professional education. As most of the interviews were conducted in Kannada, the local language, some of the meaning might have been lost in translation. In addition to this, the primary researcher in this study was inexperienced and novice in qualitative research methodology.

### Future Recommendations

Further research could involve using other qualitative methodologies such as focus group discussions (FGDs) in order to elicit richer data from the participants. Future research could explore the experiences of individuals with other mental illnesses and their experiences and perspectives of recovery in the Indian context. Exploring the opinions of other stakeholders about their perceptions of recovery could be useful in gaining a holistic perspective.

## Conclusion

In conclusion, the findings of the study show that “recovery” is understood quite literally by individuals with mental illness. Though enough individuals are trying to live their life within the limitations of their illness, this has not been recognized as recovery, as illness is still an immense part of their identity. Occupations are an important driving force that need to be utilized more effectively in the empowerment of individuals with serious mental illness. Though the idea of recovery and living a life beyond the illness might be slightly foreign to our people, there is a lot of good that can be taken from the recovery ideology. The concepts of recovery need to be applied in this context with an added consideration of its people their beliefs and sociocultural milieu.
